# Supplementary material for: A Successful Bridge Therapy Combining Hypomethylating Agents with Venetoclax for Adult Patients with Newly Diagnosed or Relapsed/Refractory Acute Myeloid Leukemia
Source: Cancers (Basel). 2023 Mar 8;15(6):1666. doi: 10.3390/cancers15061666 (PMC10046472; doi:10.3390/cancers15061666)
Supplement: Supplementary file 1 [file cancers-15-01666-s001.zip › cancers-2238498-supplementary.pdf]

Table S1. Univariate and multivariate analyses for OS, RFS, CIR and NRM in R/R-AML

	OS			RFS			CIR			NRM		
	Univariate	Multivariate		Univariate	Multivariate		Univariate	Multivariate		Univariate	Multivariate	
	p-value	*p*-value	HR [95% CI]	*p*-value	*p*-value	HR [95% CI]	*p*-value	*p*-value	HR [95% CI]	*p*-value	*p*-value	HR [95% CI]
Age at HCT	0.421	-	-	0.331	-	-	0.106	-	-	0.400	-	-
Sex	0.720	-	-	0.914	-	-	0.570	-	-	0.600	-	-
VEN-HMA cycle (>3 or ≤3)	0.998	-	-	0.137	-	-	0.265	-	-	0.999	-	-
Prior HCT (Yes or no)	0.185	-	-	0.184	-	-	0.670	-	-	**0.025**	0.316	2.673 [0.390-18.300]
ELN risk group (Poor or others)	0.114	**-**	**-**	0.242	-	-	0.571	-	-	0.370	-	-
HCT-CI (≥3 or <3)	**0.006**	0.054	3.225 [0.980-10.620]	**0.026**	0.166	2.178 [0.724-6.552]	0.740	0.977	1.023 [0.215-4.858]	**0.025**	0.067	8.459 [0.844-84.820]
Response at HCT (No or response)	**0.001**	0.056	4.356 [0.964-19.680]	**0.002**	**0.045**	**4.528** [1.028-19.940]	0.094	**0.024**	10.420 [1.359-79.930]	0.240	0.535	2.166 [0.189-24.870]
Conditioning (RIC or MAC)	0.314	-	-	0.105	-	-	0.310	-	-	0.450	-	-
Donor type (Others or matched)	0.720	-	-	0.247	-	-	0.290	-	-	0.640	-	-

OS, overall survival; RFS, relapse-free survival; CIR, cumulative incidence of relapse; NRM, non-relapse mortality; HCT, hematopoietic stem cell transplantation; R/R, relapse/refractory; ELN, European Leukemia Net; HCT-CI, hematopoietic cell transplantation-comorbidity index; RIC, reduced-intensity conditioning; MAC, myeloablative conditioning;
